# Dynamics and monitoring of insecticide resistance in malaria vectors across mainland Tanzania from 1997 to 2017: a systematic review

**DOI:** 10.1186/s12936-019-2738-6

**Published:** 2019-03-26

**Authors:** Deokary Joseph Matiya, Anitha B. Philbert, Winifrida Kidima, Johnson J. Matowo

**Affiliations:** 1 Dar es Salaam University College of Education (DUCE), P.O. Box 2329, Dar es Salaam, Tanzania; 2 University of Dar es Salaam (UDSM), P.O. Box 35064, Dar es Salaam, Tanzania; 3 Kilimanjaro Christian Medical University College (KCMUCo), P.O. Box 2240, Moshi, Tanzania

**Keywords:** *Anopheles funestus*, *Anopheles gambiae*, Insecticides, Resistance, Malaria, Vector control, Tanzania

## Abstract

**Background:**

Malaria still claims substantial lives of individuals in Tanzania. Insecticide-treated nets (ITNs) and indoor residual spray (IRS) are used as major malaria vector control tools. These tools are facing great challenges from the rapid escalating insecticide resistance in malaria vector populations. This review presents the information on the dynamics and monitoring of insecticide resistance in malaria vectors in mainland Tanzania since 1997. The information is important to policy-makers and other vector control stakeholders to reflect and formulate new resistance management plans in the country.

**Methods:**

Reviewed articles on susceptibility and mechanisms of resistance in malaria vectors to insecticides across mainland Tanzania were systematically searched from the following databases: PubMed, Google scholar, HINARI and AGORA. The inclusion criteria were articles published between 2000 and 2017, reporting susceptibility of malaria vectors to insecticides, mechanisms of resistance in the mainland Tanzania, involving field collected adult mosquitoes, and mosquitoes raised from the field collected larvae. Exclusion criteria were articles reporting insecticide resistance in larval bio-assays, laboratory strains, and unpublished data. Reviewed information include year of study, malaria vectors, insecticides, and study sites. This information was entered in the excel sheet and analysed.

**Results:**

A total of 30 articles met the selection criteria. The rapid increase of insecticide resistance in the malaria vectors across the country was reported since year 2006 onwards. Insecticide resistance in *Anopheles gambiae* sensu lato (s.l.) was detected in at least one compound in each class of all recommended insecticide classes. However, the *Anopheles funestus* s.l. is highly resistant to pyrethroids and DDT. Knockdown resistance (*kdr*) mechanism in *An. gambiae* s.l. is widely studied in the country. Biochemical resistance by detoxification enzymes (P450s, NSE and GSTs) in *An. gambiae* s.l. was also recorded. Numerous P450s genes associated with metabolic resistance were over transcribed in *An. gambiae* s.l. collected from agricultural areas. However, no study has reported mechanisms of insecticide resistance in the *An. funestus* s.l. in the country.

**Conclusion:**

This review has shown the dynamics and monitoring of insecticide resistance in malaria vector populations across mainland Tanzanian. This highlights the need for devising improved control approaches of the malaria vectors in the country.

## Background

For about two decades, African countries have been intensifying malaria vector control activities. Insecticide-treated nets (ITNs/LLINs) and indoor residual spray (IRS) have been scaled up as major tools for malaria vector control. This has resulted in a substantial reduction in malarial morbidity and mortality [1, 2]. However, the rapid expansion of insecticide resistance observed in major African malaria vectors, *Anopheles gambiae* s.l. and *Anopheles funestus* s.l. could jeopardize this achievements [2, 3]. This has been further accelerated by the use of similar insecticide classes against crop and livestock pests, thus, exerting more selection pressure to the vectors and hence worsening the situation [4]. Insecticide resistance monitoring in malaria vectors has been carried out in most of the malaria endemic countries [4]. Many countries in Africa have reported insecticide resistance in the main malaria vectors to at least two of all four major insecticide classes (pyrethroids, organochlorine, carbamates, and organophosphates) recommended for vector control. Of all the classes of insecticides, high levels of resistance have been recorded against pyrethroids, which is the only class of insecticides used in ITNs/LLINs [2, 3].

In the mainland Tanzania, efficacy and effectiveness trials of ITNs against malaria vectors were conducted between early 1980s and late 1990s [5], followed by the national wide scaling up of ITNs that started in 1996 [6, 7]. IRS programmes in the country particularly in the North Western zone around the Lake Victoria, commenced in 2007. Those programmes started with pyrethroid-based insecticide (lambdacyhalothrin) in 2007, followed with bendiocarb (carbamate) in 2011 then pirimiphos methyl (organophosphate), from 2014 to date [8–11]. Since late 1990s there have been some large-scale (having 5–29 study sites) [6, 11–16] and small-scale (having one study site) [8, 17–30] studies on insecticide susceptibility and mechanisms of resistance monitoring in malaria vectors conducted across several sentinel sites in the mainland Tanzania. Although those studies have documented insecticide resistance and/or resistance mechanisms to different insecticides, there is no collective information regarding dynamics and monitoring of insecticide resistance in the country. Therefore, we systematically analysed information from 30 published articles on the susceptibility of malaria vectors to various insecticides and resistance mechanisms across mainland Tanzania. The aim was to elucidate dynamics and monitoring of insecticide resistance in malaria vectors across the country. This information will help revealing the extent of insecticide resistance, and the major challenge in the current malaria vector control strategies. The information will be useful in directing future vector control strategies.

## Methods

### Literature search, inclusion, and exclusion criteria

Data for this review were collected from different online research databases including PubMed, Google scholar, HINARI and AGORA. The key terms used in the bibliographical searching include “Insecticide resistance/susceptibility”, “Malaria vectors”, “Anopheles”, “Tanzania”. The inclusion criteria were articles published between 2000 and 2017 on insecticide susceptibility and/or resistance mechanisms of field collected adult mosquitoes and mosquitoes raised from larval collected from the field. Reviewed articles were also restricted to studies conducted in mainland Tanzania because Zanzibar and mainland Tanzania have different levels of malaria transmission and different malaria control strategies. On the other hand, articles reporting results from larval bioassays and susceptibility of laboratory strains were excluded since they do not reflect the real field situation. Unpublished data were also excluded.

### Data handling, management, and analysis

The extracted information include year of study, vector species tested, insecticide tested, study sites, phenotypic resistance frequencies and mechanisms of resistance across different sites. This information was entered in excel sheet and cleaned i.e. checked for missing data and consistency before analysis. Descriptive statistics was used to summarize data in proportions (percentage), tables, and figures. Insecticide susceptibility was evaluated according to the 2016 World Health Organization (WHO) guideline [31]. The 2016 guideline interprets mortality rate ≥ 98% as susceptible population, mortality rate between 90 and 97% as possible resistance and mortality rate < 90% as a resistant population.

## Results

### Literature search summary

A total number of 41 articles were recovered from various databases and repositories, but only 30 met the inclusion criteria. Information such as year of study, insecticide tested, number of study sites and study themes are presented in Table 1. The data in the reviewed articles reporting insecticide susceptibility or mechanisms of resistance from 1997 to 2017 was recorded from 35 sites across mainland Tanzania.

**Table 1:** Summary of information collected from the reviewed articles that met the selection criteria

Refs.	Years^a^	Vector	Types of insecticides tested	No. of sites	Study topics
[6]	1999	*Anopheles* spp.	Permethrin and deltamethrin	5	Susceptibility
	2004	*An. gambiae* s.l.	Permethrin, deltamethrin and DDT	8	Susceptibility
[32]	2004	*An. gambiae* s.l.	–	1	*Kdr* mutation
[33]	2005/2006	*An. gambiae* s.l.	–	1	*Rdl* mutation
[34]	2007	*An. gambiae* s.l.	Permethrin	1	Resistance mechanisms and susceptibility
[35]	2009	*An. gambiae* s.l.	Deltamethrin, propoxur and malathion	2	Susceptibility
[17]	2010	*An. gambiae* s.l.	Permethrin, deltamethrin, lambdacyhalothrin, DDT and dieldrin	1	Susceptibility
[12]	2009/2010	*An. gambiae* s.l.	Permethrin, deltamethrin, lambdacyhalothrin, DDT and bendiocarb	11	Susceptibility
[8]	2011	*An. gambiae* s.l.	Permethrin, deltamethrin, lambdacyhalothrin, DDT and bendiocarb	1	Susceptibility and *kdr* mutation
[13]	2011	*An. gambiae* s.l.	Permethrin, deltamethrin, lambdacyhalothrin, DDT, propoxur and fenitrothion	14	Susceptibility
[16]	2011	*An. gambiae* s.l.	Permethrin, deltamethrin, lambdacyhalothrin, DDT, propoxur and fenitrothion	14	Susceptibility
[36]	2011	*An. gambiae* s.l.	Lambdacyhalothrin	14	Susceptibility and *kdr* mutation
[15]	2011	*An. gambiae* s.l.	Deltamethrin, DDT and bendiocarb	5	Resistance mechanisms
[37]	2011/2012	*An. gambiae* s.l.	–	1	Resistance mechanisms
[18]	2011	*An. gambiae* s.l.	Permethrin, deltamethrin, lambdacyhalothrin, DDT, bendiocarb, propoxur and fenitrothion	1	Susceptibility
[19]	2012	*An. gambiae* s.l.	Permethrin, deltamethrin, lambdacyhalothrin, DDT, bendiocarb, and pirimiphos methyl	1	Susceptibility and resistance mechanism
[20]	2013	*An. gambiae* s.l.	Permethrin, deltamethrin, DDT and bendiocarb	1	Susceptibility
[21]	2012/2013	*An. gambiae* s.l.	Permethrin	1	Susceptibility and *kdr* mutation
[30]	2009–2013	*An. gambiae* s.l.	Permethrin, deltamethrin, lambdacyhalothrin, DDT, bendiocarb, propoxur, fenitrothion and malathion	1	Susceptibility and *kdr* mutation
[14]	2013/2014	*An. gambiae* s.l.	Permethrin, deltamethrin, lambdacyhalothrin, DDT, bendiocarb, propoxur, and fenitrothion.	29	Susceptibility
[22]	2013/2014	*An. gambiae* s.l. and *An. funestus* s.l.	Permethrin, deltamethrin, lambdacyhalothrin, DDT and bendiocarb	1	Susceptibility
[23]	2014	*An. gambiae* s.l.	Permethrin and deltamethrin	1	Susceptibility
[24]	2014	*An. gambiae* s.l.	Permethrin, deltamethrin, lambdacyhalothrin, etofenprox, cyfluthrin and DDT	1	Susceptibility and *kdr* mutation
[38]	2014	*An. gambiae* s.l.	–	1	*Kdr* mutation
[25]	2013/2014	*An. gambiae* s.l.	Permethrin, deltamethrin, lambdacyhalothrin, DDT, bendiocarb and pirimiphos methyl	1	Susceptibility
[26]	2012/2014	*An. gambiae* s.l.	Permethrin, deltamethrin, DDT	1	Susceptibility
[11]	2015	*An. gambiae* s.l.	Permethrin, deltamethrin, DDT, bendiocarb, and pirimiphos methyl	20	Susceptibility
[27]	2015/2016	*An. gambiae* s.l. and *An. funestus* s.l.	Permethrin DDT, bendiocarb, and pirimiphos methyl	1	Susceptibility
[28]	2015/2016	*An. gambiae* s.l.	Permethrin, deltamethrin, lambdacyhalothrin, DDT, dieldrin, propoxur, bendiocarb, pirimiphos methyl and malathion	1	Susceptibility
[29]	2015/2016	*An. funestus* s.l.	Permethrin, deltamethrin, lambdacyhalothrin, DDT, dieldrin, propoxur, bendiocarb, pirimiphos methyl and malathion	1	Susceptibility
[9]	2015–2017	*An. gambiae* s.l. and *An. funestus* s.l.	Permethrin	1	Susceptibility and *kdr* mutation

^a^Year in which mosquito collections for testing was done

**Fig. 1:**
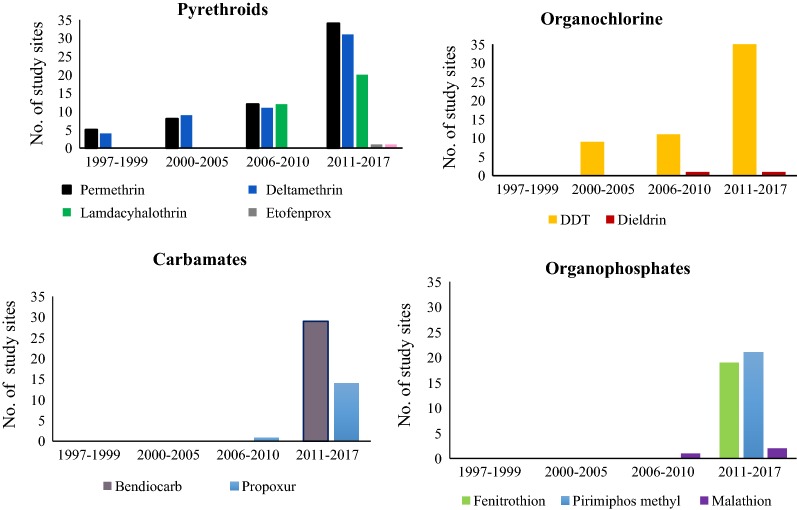
Progressive increase in the number of study sites for each class of insecticides tested from 1997 to 2017

**Fig. 2:**
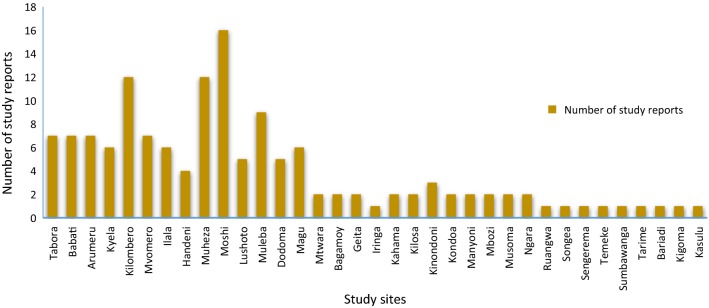
Study reports on insecticide resistance across the 35 study sites in mainland Tanzania from 1997 to 2017

There has been increasing number of study sites and reports on malaria vectors susceptibility to various insecticides and resistance mechanisms across mainland Tanzania (Fig. 1). Moreover, types of insecticide being monitored have also been increasing across the sites and in different periods of time (Fig. 1). These studies were conducted in thirty-five (35) sites. Among 35 sites, most studies were conducted in Moshi (16), Kilombero (12), Muheza (12) and Muleba (9) (Fig. 2).

### Dynamics in susceptibility status of Anopheline mosquito to insecticides across different sites in mainland Tanzania since 1997

**Fig. 3:**
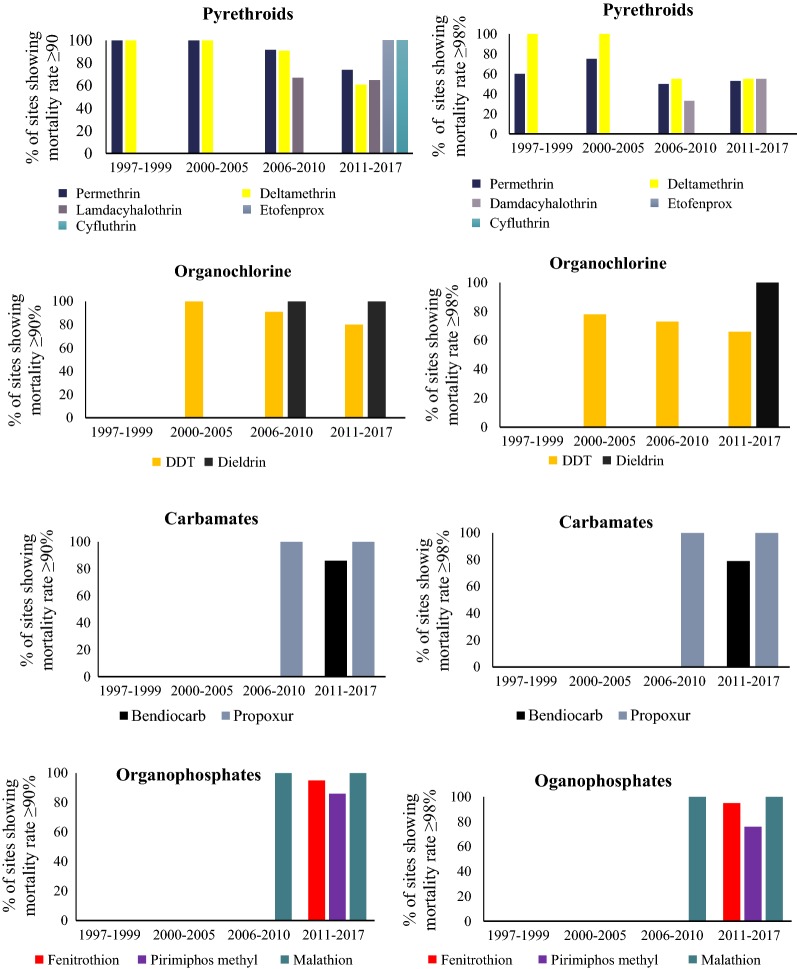
Proportion of sites showing susceptibility of *Anopheles gambiae* s.l. to different classes of insecticides≥ 90% and ≥ 98% across mainland Tanzania from 1997 to 2017

**Fig. 4:**
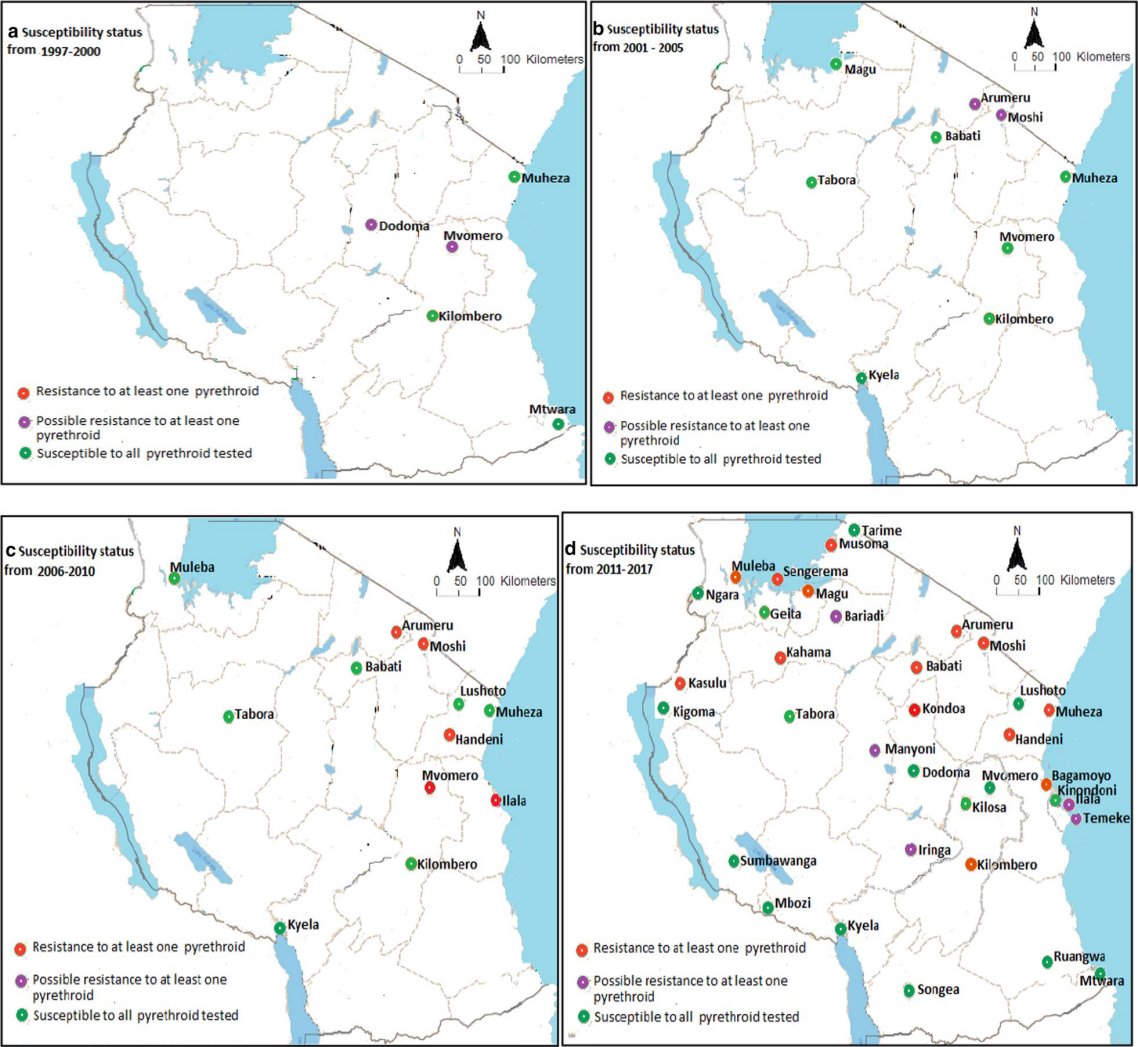
Trends of pyrethroids resistance in *Anopheles gambiae* s.l. population across the country from 1997 to 2017. **a**–**d** Study sites for pyrethroid susceptibility tests from 1997 to 2017

**Fig. 5:**
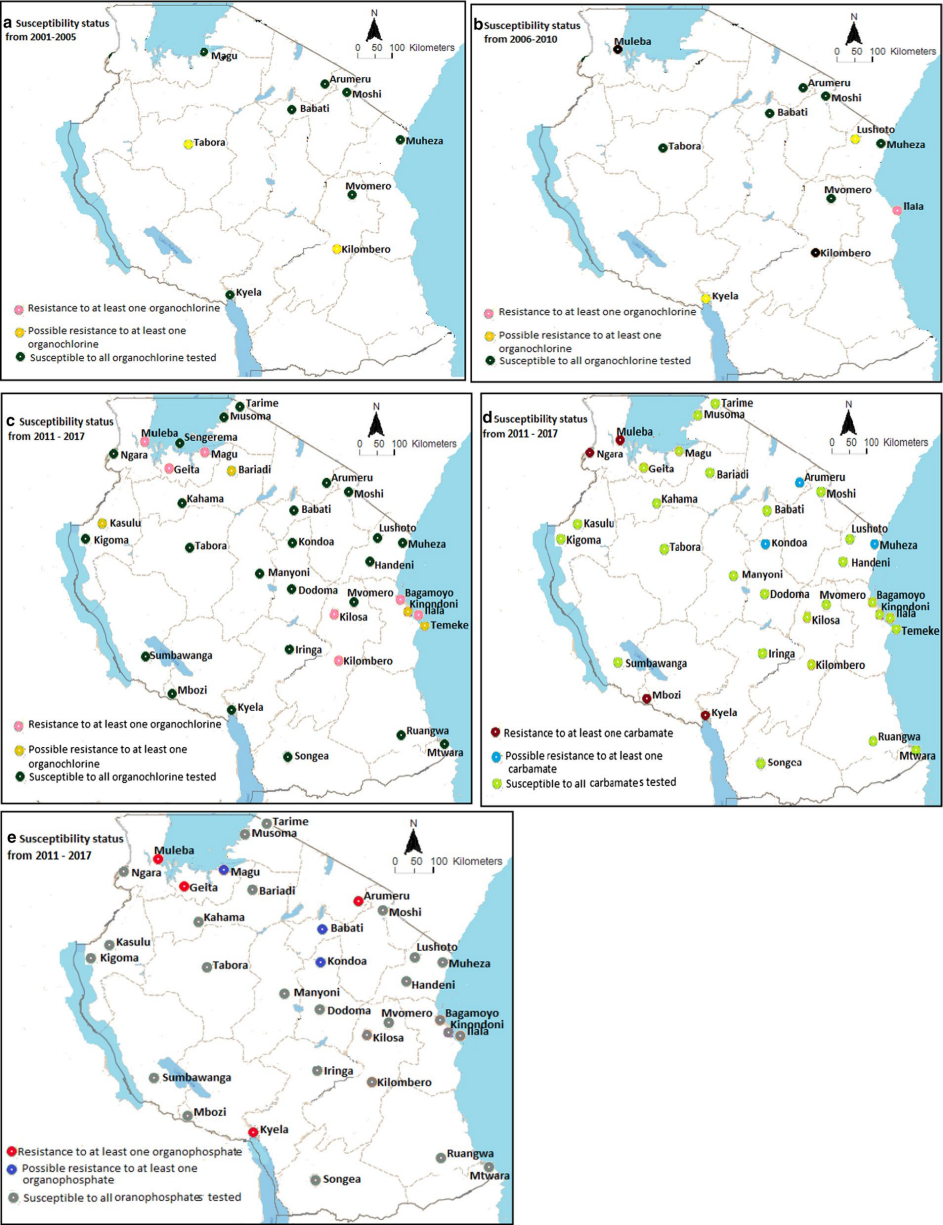
The susceptibility status of *Anopheles gambiae* s.l. to DDT, carbamates and organophosphates across mainland Tanzania from 2001 to 2017. **a**–**c** Study sites for DDT susceptibility tests from 2001to 2017; **d** Study sites for carbamates susceptibility tests from 2011 to 2017; **e** Study sites for organophosphates susceptibility tests from 2011 to 2017

Generally, from the 30 reviewed articles, sites reporting full susceptibility of *An. gambiae* s.l. to insecticides in the country have been decreasing since 1997 (Fig. 3). The decreasing pattern of mosquito susceptibility is clearly shown across pyrethroids (permethrin, deltamethrin, lambdacyhalothrin) used in ITNs and IRS in the mainland Tanzania (Fig. 4; Table 2), and DDT (Fig. 5). Before the year 2000, possibility of resistance to permethrin was observed in 40% (2/5) of sites, Dodoma and Mvomero. Between 2001 and 2005, possibility of permethrin resistance was observed in 22% (2/9) of sites, Arumeru and Moshi.

**Table 2:** Summary of the *An. gambiae* s.l. mortality rates to insecticides calculated from the pooled mosquito data since 2000

Insecticide	2000–2005			2006–2010			2011–217		
	N	n	% Mortality	N	n	% Mortality	N	n	% Mortality
Permethrin	2817	2769	98^S^	1970	1789	91^PR^	10,405	7609	73^R^
Deltamethrin	1701	1699	99.9^S^	1788	1711	96^PR^	10,995	8202	75^R^
Lambdacyhalothrin	–	–	–	1946	1709	88^R^	10,124	6329	63^R^
Etofenprox	–	–	–	–	–	–	570	536	94^PR^
Cyfluthrin	–	–	–	–	–	–	530	492.9	93^PR^
DDT	2334	2277	98^S^	1868	1818	97^PR^	12,739	12,121	95^PR^
Dieldrin	–	–	–	124	120	97^PR^	816	814	99.8^S^
Bendiocarb	–	–	–	–	–	–	4430	4227	95^PR^
Propoxur	–	–	–	–	–	–	3886	3872	99.6^S^
Fenitrothion	–	–	–	–	–	–	3516	3512	99.9^S^
Pirimiphos methyl	–	–	–	–	–	–	3054	3012	99^S^
Malathion	–	–	–	–	–	–	1316	1316	100^S^
Total	6852			8096			62,381		

*N* number of tested mosquitoes, *n* number of dead mosquitoes, *S* Susceptible, *PR* possibility of resistance, *R* resistance

Between 2006 and 2010, *An. gambiae* s.l. was reported to be resistant to permethrin, deltamethrin, lambdacyhalothrin, and DDT. The proportions of the sites where the resistance was observed for the first time for permethrin, deltamethrin, lambdacyhalothrin, and DDT were 8% (1/12), 9% (1/11), 33% (4/12) and 9% (1/11), respectively. Resistance to permethrin and lambdacyhalothrin was recorded in Moshi, deltamethrin and DDT in Ilala while resistance to lambdacyhalothrin alone was recorded in Arumeru, Handeni, and Mvomero.

There was an increase in monitoring sites of insecticides resistance from 2011 to 2017, where *An. gambiae* s.l. resistance to pyrethroids and DDT was frequently reported. Proportions of sites reporting *An. gambiae* s.l resistance to permethrin, deltamethrin, lambdacyhalothrin, and DDT by 2017 were 27% (9/33), 39% (12/31), 35% (7/20), and 20% (7/35), respectively. Additionally, carbamates and organophosphates susceptibility monitoring in the country started during this period (Fig. 1). The proportions of sites reporting *An. gambiae* s.l. resistance to bendiocarb (carbamate), fenitrothion, and pirimiphos methyl (organophosphates) were 14% (4/29), 5% (1/19) and 14% (3/21), respectively (Fig. 5).

Resistance to at least one pyrethroids compound was recorded in 14/35 sites (Fig. 4d). Furthermore, resistance to DDT alone was recorded in Kilosa and Ilala [11, 13], while resistance to carbamates alone was recorded in Mbozi and Ngara [11]. Multiple resistance was also observed where resistance to pyrethroids and DDT was observed in Kilombero, Bagamoyo and Magu [11, 16, 22], resistance to pyrethroids and organophosphates was observed in Arumeru [11], resistance to DDT and organophosphates was observed in Geita [11], while resistance to carbamates and organophosphates was observed in Kyela [14]. Resistance to all four classes of insecticides was recorded in Muleba [11].

**Fig. 6:**
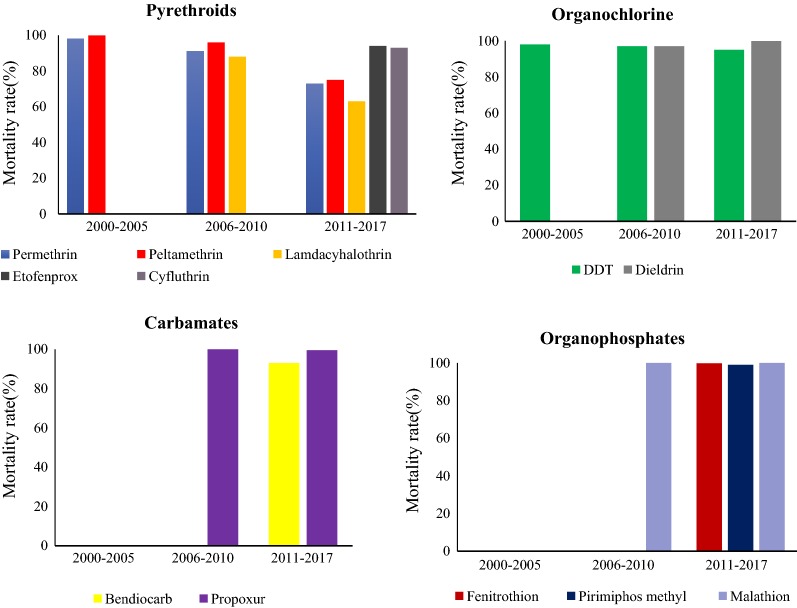
Trends of susceptibility status of *Anopheles gambiae* s.l. to insecticides calculated from pooled data of tested mosquitoes across mainland Tanzania from 2000 to 2017

Generally, the susceptibility of *An. gambiae* s.l. to public health insecticides (permethrin, deltamethrin, lambdacyhalothrin, and DDT) between years 2000 and 2017 has shown a decreasing trend (Table 2 and Fig. 6). During this period, the number of sites for susceptibility monitoring increased dramatically.

**Table 3:** Proportion of sites showing resistance of *An. funestus* s.l. to various insecticides in Tanzania from 1997 to 2017

Insecticide class	Insecticide	No. of study sites with mortality rates < 90%/Total No. of study sites (%)			
		1997–2000	2001–2005	2006–2010	2011–2017
Pyrethroids	Permethrin	–	–	–	2/3 (66.7%)
	Deltamethrin	–	–	–	1/1 (100%)
	Lambda cyhalothrin	–	–	–	1/1 (100%)
Organochlorine	DDT	–	–	–	2/2 (100%)
	Dieldrin	–	–	–	1/2 (50%)
Carbamates	Bendiocarb	–	–	–	0/2 (0.0%)
	Propoxur	–	–	0/1(0.0%)	0/1 (0.0%)
Organophosphates	Pirimiphos methyl	–	–	–	0/2 (0.0%)
	Malathion	–	–	0/1 (0.0%)	0/1 (0.0%)

There have been few studies on susceptibility of *An. funestus* s.l. to insecticides in mainland Tanzania. These studies were conducted between 2006 and 2017 in Kilombero, Muheza and Muleba [9, 22, 27, 29] (Table 3). High level of resistance was recorded against pyrethroids and DDT in Kilombero [22, 29] and only pyrethroids alone in Muleba [9]. On the contrary, full susceptibility to all tested insecticides was reported in Muheza [27, 35].

### Insecticide resistance mechanisms

In mainland Tanzania studies investigating insecticide resistance mechanisms in *An. gambiae* s.l. started early in 2000s [32]. Subsequently, there have been some large [11, 36] and small-scale studies [8, 9, 15, 21, 24, 28, 30, 33, 34, 37, 39] conducted in the country as summarized in Table 4 and 5. Knock down resistance (*kdr*) mutations conferring resistance to pyrethroids and DDT is frequently reported in the country. Both *kdr* Eastern and Western variants (L1014S) and (L1014F) respectively, were confirmed in *An. gambiae* sensu stricto (s.s.) and *Anopheles arabiensis*, the sibling species of *An. gambiae* s.l. Their allelic frequencies and geographical distribution are summarized in Table 4. The *kdr* East mutation is predominant in *An. gambiae* s.s. while the *kdr* West in *An. arabiensis* (Table 4). Highest allelic frequencies of L1014S in mainland Tanzania were recorded in North-Western, particularly in Muleba (range, 24–100%). This was followed by the Eastern zone (range, 33–84%), while no L1014S mutations were recorded in North-Eastern highlands.

**Table 4:** Distribution of *kdr* mutation in the major malaria vectors across Tanzania from 1997 to 2017

Geographical zones	Species	*kdr*	Frequency (%)				Refs.
			1997–2000	2001–2005	2006–2010	2011–2017	
*Eastern zone* Dar es salaam, Muheza, Handeni, Bagamoyo and Mvomero	*An. gambiae* s.s.	L1014S	–	–	–	33–84	[15, 36]
		L1014F	–	–	–	0.07	
	*An. arabiensis*	L1014S	–	–	–	0.6–4	
		L1014F	–	–	–	6–41	
*North-Eastern zone* Moshi and Babati	*An. gambiae* s.s.	L1014S	–	–	–	–	[30, 32, 36]
		L1014F	–	–	–	–	
	*An. arabiensis*	L1014S	–	–	–	–	
		L1014F	–	0.016	–	0.08–12	
*North-Western zone* Muleba and Sengerema	*An. gambiae* s.s.	L1014S	–	–	–	24–100	[8, 9, 24, 36]
		L1014F	–	–	–	7.7–9	
	*An. arabiensis*	L1014S	–	–	–	1.2	
		L1014F	–	–	–	38

**Table 5:** Mechanisms of insecticide resistance detected in *Anopheles gambiae* s.s. and *Anopheles arabiensis* populations

Species	Insecticide class	Insecticide	Mechanisms of resistance			Refs.
			Target site genes	Detoxification genes	Cuticular	
*An. arabiensis*	Pyrethroids	Deltamethrin and permethrin	L1014S	CY6P3, CYP9J4, CYP9J5, and CYP6P1. High activity of Oxidase and esterase enzymes	CPAP3-A1b, CPR5 and CPLCG5	[15, 36, 38]
*An. arabiensis*	Pyrethroids	Permethrin	L1014S	CYP4G16, ABC transporter and high activity of Oxidase and esterase enzyme		[9, 24, 28, 34, 37]
*An. gambiae* s.s. and *An. arabiensis*	Pyrethroids	Lambdacyhalothrin	L1014S L1014F	Presence and high activity of oxidase and esterase enzyme		[9, 24, 28, 34, 36]
*An. gambiae* s.s.	Pyrethroids	Permethrin	L1014S L1014F			[9]
*An. gambiae* s.s.	Organochlorine	DDT	L1014S	Over transcription of CYP4J10, CY6P3, multicopper oxidase and sulfotransferase genes and high activity of GST enzymes		[11, 39]
*An. arabiensis*	Organochlorine	Dieldrin	RDL, over transcription GABA receptor genes			[15, 33]
*An. arabiensis*	Organophosphates		Over transcription of acetylcholinesterase genes	High activity of esterase enzymes		[11, 15]
*An. arabiensis*	Carbamates		Over transcription of acetylcholinesterase genes	Presence and high activity of oxidase		[11, 15, 28]
*An. arabiensis*	Neonicotinoids		Over transcription of nicotinic acetylcholine receptor genes			[15]
*An. gambiae* s.s.	Different classes		–		CPLCG4, CPLCG5, CPLCG15 and CPR131	[39]

Similarly, high frequency of L1014F allele was recorded in Eastern zone ranging from 6% to 41%, followed by North-Western zone (38%), lastly in North-Eastern highlands (8–12%). The *Rdl* mutation that confers resistance to dieldrin was recorded by one study conducted in Moshi [33]. Over transcription of gamma-Amino butyric acid (GABA) receptor genes were also reported [15]. No *Ace*-1gene mutation conferring resistance to carbamates and organophosphates was detected despite over transcription of acetylcholinesterase [15].

Studies to investigate metabolic mechanisms of resistance in *An. gambiae* s.l. were done by three different methods including the use of synergists [19, 28], biochemical assays [11, 34] and molecular methods [15, 37, 39]. Synergy studies involving pre-exposing mosquitoes to piperonyl butoxide (PBO) and triphenyl phosphate (TPP)/*S*,*S*,*S*-tributyl phosphorotrithioate (DEF) respectively before WHO insecticide bioassays have confirmed involvement of P450 monooxygenases (P450s) and nonspecific esterases (NSE) on *An. gambiae* s.l. pyrethroids resistance in Kilombero [28] and Muleba [19]. Biochemical assays of detoxification enzymes in mosquitoes have evidenced significant elevation of detoxifying enzymes activities in various sites in the country: P450 s alone in Bagamoyo and Kondoa [11] P450s and nonspecific esterases (NSE) in Moshi [34], glutathione-*S*-transferases (GSTs) in Kahama and Kyela, and all three (3) enzymes in Arumeru [11].

Molecular mechanisms of metabolic resistance have also been documented in the country. The over transcription of P450s gene family: CYP6P3 and CYP9J5 in *An. arabiensis* was recorded in Dar es Salaam; associated with DDT and pyrethroid resistance. Over transcription of CYP9J4 and CYP6P1 was recorded in Hai [15] and CYP4G16 and ABC transporter in Moshi [37]; associated with in *An. arabiensis* pyrethroids resistance. Over transcription of P450 s, CYP4J10 and CY6P3 in *An. gambiae* s.s. was also recorded in Dar es Salaam; associated with DDT resistance [39]. Furthermore, over transcription of CPAP3-A1b, CPR5 and CPLCG5 genes encoding cuticle protein which are associated with insecticide resistance in *An. gambiae* s.l. was also recorded in Dar es Salaam (urban area) and in Hai (agricultural area) [15, 39].

## Discussion

This review aimed at demonstrating dynamics and monitoring of insecticide resistance in malaria vectors in mainland Tanzania between 1997 and 2017. A total of 30 published articles reporting results from 35 study sites were reviewed. Most studies were conducted from Eastern, North-Eastern highlands and North-Western parts of the mainland Tanzania. Commencing from late 1990s, number of study sites and types of insecticide classes for controlling disease vectors have been expanding. Similarly, the increase in resistance of malaria vectors to widely studied insecticides (pyrethroids and DDT) was observed. Whereas, most studies on organophosphates and carbamates started after the year 2010. The wide spread of resistance observed between 2011 and 2017 in mainland Tanzania is a result of development of insecticide resistance in malaria vectors in areas which have previously recorded full susceptibility. For instance, 50% (7/14) and 43% (3/7) of study sites, which have recorded *An. gambiae* s.l. resistance to pyrethroids and DDT respectively by 2017, showed high susceptibility in studies conducted before year 2009.

Moreover, an increasing number of resistance monitoring sites over time has also detected new sites with insecticide resistance in malaria vectors. The mainland Tanzania consists of about 187 districts [40] and insecticide resistance monitoring studies have so far been conducted in 18% of total districts. Increasing the number of study sites and regular monitoring of susceptibility of mosquitoes to insecticides is needed to give a broader picture of the trend of insecticide resistance status in the country.

By 2017, *An. gambiae* s.l. resistance to all four recommended classes of insecticides used in malaria vector control had been reported from various parts of the country. Efficacy of pyrethroids and DDT seems to be greatly threatened by the observed resistance. Although DDT is not currently used in vector control, susceptibility status of malaria vectors to this insecticide should be known because of its cross resistance with pyrethroids. The observed DDT resistance might be due to the cross resistance with pyrethroids or historical use of DDT in the country. On the other hand, susceptibility studies on *An. funestus* s.l. show they are highly resistant to pyrethroids and DDT while highly susceptible to carbamates and organophosphates. This implies that carbamates and organophosphates remain good candidate insecticides for controlling *An. funestus* s.l. in the country.

In this review, high frequencies of both west and east African *kdr* mutations in *An. gambiae* s.l. were observed in Eastern and North-Western parts of the country. Similarly, pyrethroids and DDT resistance had been detected in the same areas. This is due to the cross resistance pattern existing among these compounds that share the common target site that is voltage gated sodium channels (VGSC) of insect nerve cell [41]. However, *kdr* mutations are strongly associated with DDT and less associated with pyrethroids resistance [42]. Different studies in the country have recorded resistance to either pyrethroids alone, DDT alone or resistance to more than one compound with unrelated target sites (e.g. pyrethroids or DDT with organophosphates or carbamates). This situation might be triggered by metabolic resistance of which cross resistance is difficult to generalize [43]. For instance, P450 s enzymes in some cases have shown specificity by acting differently in compounds within the same insecticide class [43–45]. In other cases P450s enzymes have metabolized compounds between different classes of insecticides [44, 45]. Moreover, a metabolic resistance study conducted by Kisinza et al. [11] across the country has shown elevation of detoxification enzymes associated with different classes of insecticides.

Areas, which have recorded high *kdr* frequencies in this review, are dominated by *An. gambiae* s.s., while other areas with high phenotypic resistance and low or no *kdr* mutations are being dominated by *An. arabiensis* [8, 28, 30, 32, 36]. This suggests that *An. arabiensis* resistance to insecticides mostly depend on metabolic path-way. According to Rusell et al. [46], *An. arabiensis*, is the predominant species of the *An. gambiae* s.l. over *An. gambiae* s.s. in most areas of the country. Their exophilic and exophagic behavior have reduced their mortality from exposure of the indoor insecticide applications of LLINs and IRS, thus, increase in their dominance. This increase dominance of *An. arabiensis* over *An. gambiae* s.s. in the country should be coupled with deployment of new tools for reducing residual malaria transmission.

There are limited studies on molecular mechanisms of metabolic insecticide resistance in *An. gambiae* s.l. in Tanzania. A study by Nkya et al. [15] revealed over transcription of several genes in *An. arabiensis* as compared to the susceptible laboratory colony, mostly in agricultural areas. The most significantly over transcribed genes in *An. arabiensis* were P450s: CYP6P3 and CYP9J5 associating with pyrethroid and DDT resistance in Dar es Salaam (urban); and CYP9J4 and CYP6P1 genes in associating with pyrethroid resistance in Hai district (agricultural areas) [15]. Conversely, another study by Matowo et al. [37] conducted in Moshi district (agricultural areas) near Hai district has recorded over transcription of different genes in *An. arabiensis,* which include over transcription of CYP4G16 and ABC transporter genes in *An. arabiensis* associating with pyrethroids resistance [37]. Nevertheless, over transcription of CYP4J10 and CY6P3 genes in *An. gambiae* s.s. associating with DDT resistance was also observed in urban areas [39]. These findings are contrary to studies conducted elsewhere in Africa where over transcription of CY6P3, CYP6M2, CYP6Z1, CYP6Z2 and Cyp9K1 genes associating with pyrethroid or DDT resistance was observed [47–52]. This indicates that different genes, depending on the environment in which mosquitoes are exposed, might mediate metabolic resistance in *An. gambiae* s.l. In another study, over transcription of CPAP3-A1b genes encoding cuticle protein was also recorded in agricultural areas in the country and were associated with pyrethroid resistance [15]. Over transcription of these genes was also recorded elsewhere in West Africa [53]. There is a need to explore further involvement of other metabolic and cuticular resistance genes associated with pyrethroid resistance in Tanzania where pyrethroid LLINs is scaled up as the main malaria vector control tool. So far, no studies in the country that have reported mechanisms of resistance in the *An. funestus* s.l.

There are several factors, which might have contributed to the intensified development, spread and variations in malaria vectors resistance to various insecticides across different sites in the country. These factors include historical use of pesticides, contemporary use of pesticides in agriculture, non-pesticides pollutants and insecticides use in vector control.

### The historical role of pesticides usage in the development of insecticide resistance in malaria vectors

Between 1940s and 1960s malaria vector control by IRS and aerial spray of organochlorines (DDT, dieldrin and lindane) were conducted in Dar es Salaam [5]. As part of global malaria eradication campaign between 1950 and 1960, pilot projects of IRS with DDT and dieldrin were conducted in Pare-Taveta border. Some other low scale IRS activities were conducted in selected areas from the five region of mainland Tanzania [5]. From 1970s to 1980s organochlorines pesticides were also vastly donated to the country for agricultural pest control [54]. Areas in the country with the known history of massive utilization of these pesticides include: Dar es Salaam, Lake Victoria basin and Moshi [55]. Use of DDT and other persistent organochlorine insecticides were banned in the country for public health use in 1980s and for agricultural use in 1997 [56]. This was due their hazardous effect on human beings, biodiversity and environmental concerns [56]. Following banning, there were stockpiles of improperly kept pesticides which had contaminated the environment around storage sites [54]. Some of the nine major unattended storage sites are located in the six regions of the main-land Tanzania [56]. Several studies conducted around some storage sites and other areas which have largely utilized these pesticides, have detected their residuals in water, soil and plants [24, 55–61]. These insecticide residuals may also be exposed to mosquitoes by leaching to their breeding sites [24]. Currently, there has been a rapid development of DDT and pyrethroids resistance in some parts of the Lake Victoria zone after deployment of pyrethroids intervention (LLINs and IRS). This might be due to the existence of small population of resistant mosquitoes from DDT residuals exposures followed by pyrethroid exposures. DDT and pyrethroids share the same target site, hence cross resistance between these insecticides [24]. However, the effect of the DDT contamination around former storage sites on malaria vector insecticide resistance has not been established. Therefore, investigations of pyrethroids and DDT resistance around those former pesticide storage sites are needed, to evaluate their contribution to the current DDT and pyrethroids resistance in malaria vectors.

### The role of contemporary usage of pesticide in agriculture in the development of insecticide resistance in malaria vectors

Importation of pesticides increased enormously in Tanzania after trade liberalization of agricultural inputs in 1990s [62]. Pesticides imports increased from about 500 to 12,000 metric tons between 2000 and 2014, respectively [63]. More than 80% of pesticides have been used for agricultural and veterinary purposes [54]. Large amount of pesticides have been used in the country to control pests in horticulture, floriculture, cotton, cashew nuts, coffee, sugar cane, legumes and rice cultivation [64]. Pesticides frequently used in the country include: pyrethroids (alpha cypermethrin, deltamethrin, lambdacyhalothrin and permethrin), organophosphates (chlorpyrifos, dimethoate, profenofos, pirimiphos methyl, diazinon, malathion, fenitrothion and glyphosate) organochlorines (endosulfan, chlorothalonil) carbamates (carbofuran, carbaryl, aldicarb and mancozeb) [20, 62–71]. These insecticide classes are the same as ones used in public health vector control, hence accelerating selection of resistance in malaria vectors. Accordingly, several studies in Africa have shown an association between agricultural practices and insecticides resistance in malaria vectors, reviewed in [72]. Additionally, similar observations have been reported in Tanzania [15, 20, 24, 37, 39]. Studies on molecular mechanisms of resistance conducted in the areas with intensive agricultural practice in Lower Moshi and Hai have reported over transcription of several genes associated to pyrethroid resistance compared to non-agricultural areas [15, 37]. Moreover, a study by Kisinza et al. [11] conducted in Arumeru, has shown the high resistance of *An. gambiae* s.l. to pyrethroids and pirimiphos methyl (organophosphate). This *An. gambiae* s.l. resistance to pyrethroids and pirimiphos methyl might be a result of the long time large scale use of pyrethroids and organophosphates used in agricultural pest control in Arumeru [67, 69, 73–76].

### The role of non-pesticide pollutants in the development of insecticide resistance in malaria vectors

Mosquitoes breeding sites are exposed to various pollutants from different human activities. *Anopheles gambiae* s.l., which normally breed in clean water, have currently been found in polluted breeding sites of the urban and rural areas in Tanzania [77, 78]. Breeding sites of mosquitoes have been found to be contaminated with several pollutants including organic pollutants (sewage, rotting plants, domestic and animal wastes and other industrial organic chemicals); also inorganic pollutants such as heavy metals (Zn, Au, Mn, Hg, Pb, Cu, Fe, Cd and Co) [79, 80] and others (industrial and agrochemicals). Pollutants affect the physical–chemical parameters of breeding sites, consequently may affect mosquitoes’ genetic makeup [81–84]. These physical–chemical parameters include pH, conductivity, turbidity, total dissolved solids (TDS) and total suspended solids (TSS), Na^+^, Ca^2+^, K^+^, NH_4_^+^, SO_4_^2−,^ PO_4_^2−^, NO_3_^−^, NO2^−^, H_2_O_2_, Cl^−^ and F^−^ [78, 81]. Limited studies exist on the influence of those pollutants to the development of insecticide resistance in malaria vectors [79–81, 84, 85]. Some pollutants or the change in the levels of physical–chemical parameters in mosquitoes’ breeding sites have shown toxic effect to larval stages [80, 86]. Mosquito larvae surviving such environment have shown tolerance against the effect of those toxic pollutants and subsequently develop insecticide resistance in adult stages [84, 87].

Vividly, a study by Tene Fossog et al. [86] has demonstrated that *Anopheles coluzzi* larvae developed tolerance after being exposed to the lethal dose of NH_3_. Moreover, other studies have shown that Anopheles larvae exposed to heavy metals or disinfectants (H_2_O_2_ and/or soap) developed insecticide tolerance in their adult stages [80, 87]. Some pollutants and levels of physical–chemical parameters in *An. gambiae* s.l. breeding sites have shown to be positively associated with their insecticide resistance [81, 83, 84, 88]. These pollutants and physical–chemical parameters include greases, oils, TDS, NH_3_, NH_4_^+^, SO_4_^2−^, PO4^2−^, NO_3_^−^, NO_2_^−^ and F^−^. A study by Emidi et al. [78] in Muheza district, Tanzania, correlating physico-chemical parameters in mosquitoes breeding sites and larval density, have reported for the first time the occurrence of *An. gambiae* s.l. in polluted habitat in rural areas. However, the influence of these pollutants to the insecticide resistance across the different ecological zones in the country is yet to be investigated.

### The role of public health insecticides use in the development of resistance in malaria vectors

The use of LLINs and IRS are major vector control strategies currently utilizing substantial amount of insecticides [89]. Scaling up of ITNs in mainland Tanzania started in the late 1990s after their efficacy and effectiveness trials between 1980s and early 1990s. Scaling up of ITNs has been going on through different programmes including: Kilombero Net Project (KINET) in 1996, Social Marketing for ITNs Project (SMITN)/Strategic Social Marketing for Expanding the Commercial Market (SMARTNET) in 1998 [6]. Others are Tanzania National Voucher Scheme (TNVS) in 2004, Under Five Catch-Up Campaign (U5CC) in 2008 and Universal Catch-Up Campaign (UCC) since 2010 [7]. Finally, Keep-Up projects started from 2013 to date [90]. From those projects ITNs coverage of one net per household raised from about 23% in 2004 to more than 91% in 2011. On the other hand, IRS scaling up in the lake zone started in 2007 with the successive replacement of insecticide classes. Insecticides used on IRS include lambdacyhalothrin since 2007, bendiocarb since 2011 and pirimiphos methyl since 2014 to date. It is estimated that, the quantity of insecticides imported in Tanzania for ITNs and IRS had approximately increased from 10 metric tons in 2007 [91–94] to 311 metric tons in 2012 [7, 91, 94]. This amount of insecticides used in both ITN and IRS is about 2 to 3% of total imported pesticides, which is relatively very small compared to 80% of total insecticide used agriculture and veterinary pest control [63]. However, mosquitoes’ contact with insecticides in ITNs and IRS might be high because these strategies target their indoor resting and blood feeding behaviour. Evidently, scaling up of ITNs and IRS activities, as well as the use of aerosols, fumigations and coils, particularly among urban populations, are strongly associated with rapid expansion of insecticide resistance in malaria vectors since 2004. The insecticide-based approaches might have exerted selection pressure on Tanzanian populations of both *An. gambiae* s.l. and *An. funestus* s.l. Indeed, *An. gambiae* s.l. has currently developed resistance to at least one compound in all four insecticides classes in Muleba. This area has a long history of utilizing both ITNs and IRS to reduce existing high malaria burden [11]. Therefore, mitigation of resistance situation in the country needs involvement of different authorities from agricultural, public health and environmental sectors in order to preserve effectiveness of insecticides for vector control.

## Conclusions

This review has provided a picture of dynamics and monitoring of insecticide resistance in major malaria vectors in mainland Tanzania, hence highlighting challenges facing the current malaria control tools in the country. Currently, *An. gambiae* s.l. has shown resistance to at least one compound of all insecticides classes used in the country for malaria control. On the other hand, *An. funestus* s.l. has shown resistance to pyrethroids and DDT while still susceptible to other classes. However, only few susceptibility studies have been carried out on this group of malaria vectors in the country. Emergence and spread of the observed insecticide resistance could be accelerated by different factors like the use of pesticides in agriculture and public health as well as environmental pollution. Therefore, mitigation of this situation needs involvement of different sectors in order to preserve effectiveness of insecticides for vector control. Data from this review may be used as basis of devising new control strategies of malaria vectors in the country.

## Abbreviations

DDT: dichlorodiphenyltrichloroethane
GABA: gamma-amino butyric acid
GSTs: glutathione-*S*-transferases
ITNs: insecticide-treated nets
*kdr*: knock down resistance
LLINs: long-lasting insecticide-treated nets
IRS: indoor residual spraying
NSE: non-specific esterases
PBO: piperonyl butoxide
TPP: triphenyl phosphate
VGSC: voltage gated sodium channels
WHO: World Health Organization
